# Baseline gut microbiome composition predicts metformin therapy short-term efficacy in newly diagnosed type 2 diabetes patients

**DOI:** 10.1371/journal.pone.0241338

**Published:** 2020-10-30

**Authors:** Ilze Elbere, Ivars Silamikelis, Ilze Izabella Dindune, Ineta Kalnina, Monta Briviba, Linda Zaharenko, Laila Silamikele, Vita Rovite, Dita Gudra, Ilze Konrade, Jelizaveta Sokolovska, Valdis Pirags, Janis Klovins

**Affiliations:** 1 Latvian Biomedical Research and Study Centre, Riga, Latvia; 2 Riga Stradins University, Riga, Latvia; 3 Faculty of Medicine, University of Latvia, Riga, Latvia; University of Minnesota Twin Cities, UNITED STATES

## Abstract

**Background:**

The study was conducted to investigate the effects of metformin treatment on the human gut microbiome’s taxonomic and functional profile in the Latvian population, and to evaluate the correlation of these changes with therapeutic efficacy and tolerance.

**Methods:**

In this longitudinal observational study, stool samples for shotgun metagenomic sequencing-based analysis were collected in two cohorts. The first cohort included 35 healthy nondiabetic individuals (metformin dose 2x850mg/day) at three time-points during metformin administration. The second cohort was composed of 50 newly-diagnosed type 2 diabetes patients (metformin dose–determined by an endocrinologist) at two concordant times. Patients were defined as Responders if their HbA1c levels during three months of metformin therapy had decreased by ≥12.6 mmol/mol (1%), while in Non-responders HbA1c were decreased by <12.6 mmol/mol (1%).

**Results:**

Metformin reduced the alpha diversity of microbiota in healthy controls (p = 0.02) but not in T2D patients. At the species level, reduction in the abundance of *Clostridium bartlettii* and *Barnesiella intestinihominis*, as well as an increase in the abundance of *Parabacteroides distasonis* and *Oscillibacter* unclassified overlapped between both study groups. A large number of group-specific changes in taxonomic and functional profiles was observed. We identified an increased abundance of *Prevotella copri* (FDR = 0.01) in the Non-Responders subgroup, and enrichment of *Enterococcus faecium*, *Lactococcus lactis*, *Odoribacter*, and *Dialister* at baseline in the Responders group. Various taxonomic units were associated with the observed incidence of side effects in both cohorts.

**Conclusions:**

Metformin effects are different in T2D patients and healthy individuals. Therapy induced changes in the composition of gut microbiome revealed possible mediators of observed short-term therapeutic effects. The baseline composition of the gut microbiome may influence metformin therapy efficacy and tolerance in T2D patients and could be used as a powerful prediction tool.

## Introduction

Type 2 diabetes (T2D) is a metabolic disease with rapidly increasing prevalence, characterized by variable etiology, clinical presentation, and consequences. Metformin has been used in clinical practice for more than 60 years [1, 2] and is currently considered as a first-choice medication for T2D treatment worldwide. Regardless of its diverse beneficial impact on health, more than 20% of patients fail to reach the glycemic target when on metformin monotherapy [3], and more than 30% experience mostly gastrointestinal (GI) side effects (SE) [4]. These results suggest that the gut microbiome is an intermediary of metformin therapy, which highlight the need for the development of precision medicine-based therapeutic approaches [5]. Because of the complex structure, compositional and functional dynamics, and the host-microbiome interaction, the microbiome has been postulated as a key component of precision medicine approaches [6]. Moreover, latest studies have suggested that metagenomics predictive tools for T2D should be specific for the age and geographical location of the population studied [7]. Several studies on the interaction between the human gut microbiome and metformin have been performed [8–12], but most of these studies used a case-control design involving patients with different diabetes duration and therapy history.

The aim of this study was to investigate the effects of metformin treatment on the taxonomical and functional profile of the human gut microbiome and to evaluate the correlation of these changes with the therapeutic efficacy and tolerance in a prospective cohort of T2D patients. Our research provides novel information on short-term effects induced by metformin with the advantage of longitudinal data, including treatment naïve patients, as well as characterizes the predictive quality of baseline microbiota composition. In addition, the growing evidence on other therapeutic targets of metformin requires more detailed information on metformin effects in non-diabetic populations, therefore, the data from the healthy cohort offer complementary value.

## Materials and methods

### Study design, sample and data collection

The study involved two longitudinal cohorts of participants: OPTIMED cohort of newly-diagnosed T2D patients (N = 50) recruited within the framework of Genome Database of Latvian population [13], and a cohort of healthy individuals (N = 35). A full list of inclusion/exclusion criteria for both cohorts can be found in the S1 Text. Informed consent was obtained from all participants at the beginning of the study. Healthy individuals received 850mg metformin twice a day for 7 days within the framework of the clinical trial (registration number: 2016-001092-74 (www.clinicaltrialsregister.eu)), while T2D patients were treated with metformin monotherapy according to therapy prescribed by an endocrinologist (individual dosage, titration, etc.). The study was carried out in accordance with the principles of the Declaration of Helsinki, and approved by the Central Medical Ethics Committee (1/19-10-22) and State Agency of Medicines of the Republic of Latvia (17–1723). The primary and secondary endpoints together with other methodological details (regarding the clinical trial involving healthy volunteers and standard operating procedures in place for stool sample collection) have been described previously [11].

**Fig 1:**
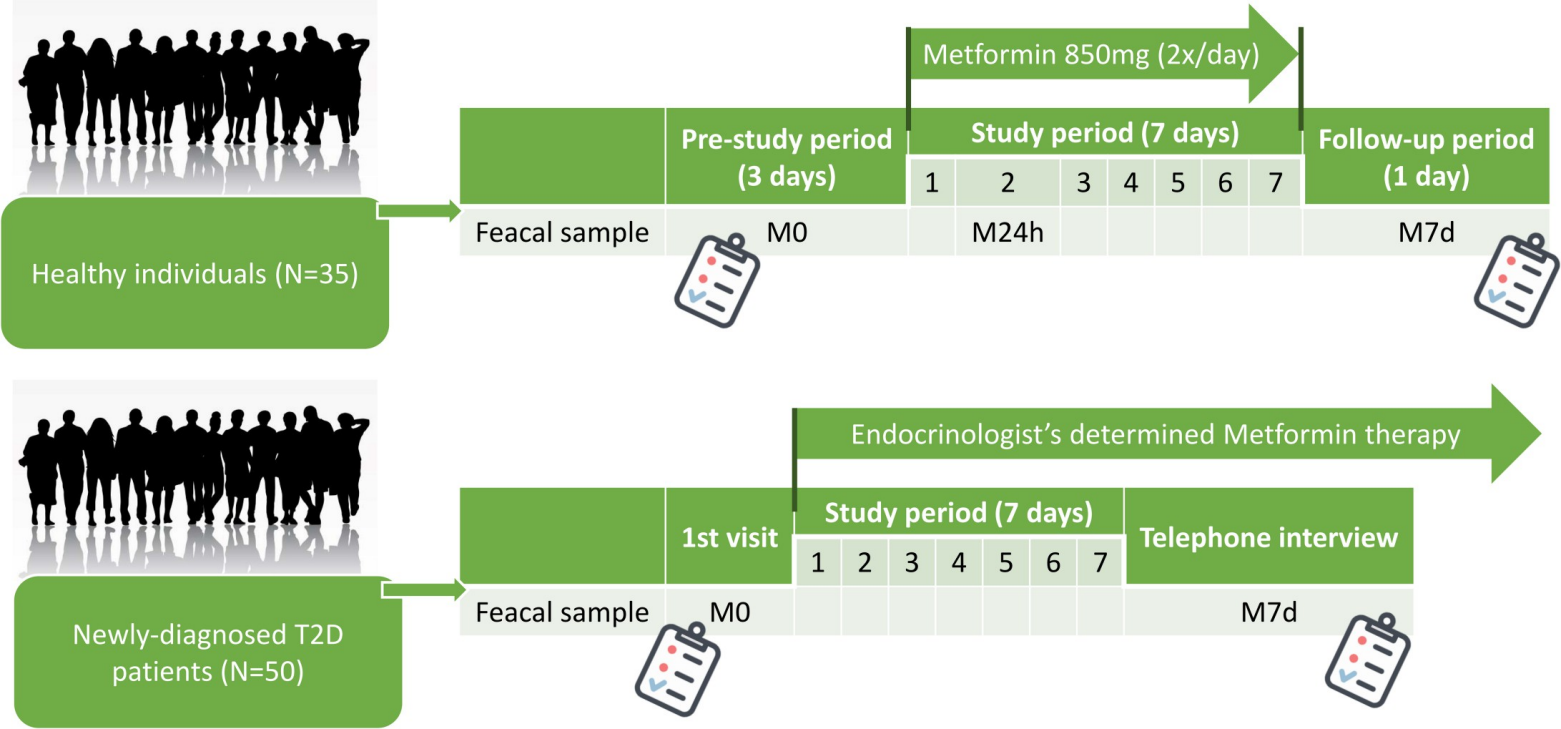
Study design depicting sample collection in both cohorts. Samples were coded as follows: M0 –before starting metformin treatment, M24h – 24 hours after first metformin dose, and M7d – 7 days after the first intake of metformin. T2D –type 2 diabetes.

Stool samples were collected in two aliquots at pre-determined time points during the study, depending on the design for each study cohort (Fig 1). Samples were coded as follows: M0 – before metformin treatment, M24h – 24 hours after the first metformin dose (only in the study group of healthy individuals), and M7d – 7 days after starting the therapy. All samples were collected by participants at home, using sterile collection tubes without buffer (collection date and time were marked). Within 24 hours participants delivered samples to the closest clinical or research laboratory where samples were frozen at −80°C (delivery time was registered).

Blood samples for biochemical/hematological analysis (conducted in a certified clinical laboratory) to evaluate inclusion/exclusion criteria and obtain relevant clinical data were collected from participants within both cohorts. Samples were collected in the fasting state before starting metformin administration. For the patient cohort, a repeated biochemical/hematological analysis was performed three months later (follow-up coded as a time point M3m).

The information on anthropometric measurements, dietary habits, and biochemical/hematological analyses was obtained before starting metformin administration. Healthy volunteers registered their diet during the metformin administration, as well as any observed SE in special questionnaires. Patients of OPTIMED cohort were interviewed via phone by their endocrinologists after the first week of metformin therapy to register possible metformin-induced SE.

For the analysis of gut microbiome mediated metformin’s therapy efficacy patients were divided into two subgroups based on the observed reduction of HbA_1c_ during three months long metformin therapy. Patients were defined as Responders if their HbA_1c_ levels had decreased by ≥12.6 mmol/mol (1%), or Non-responders if their HbA_1c_ levels had decreased by <12.6 mmol/mol (1%). This threshold has been previously established within a systematic review comparing three months long metformin therapy with placebo and used in other studies as well [14, 15].

### Sample processing and sequencing

Microbial DNA was extracted using the FastDNA Spin Kit for Soil (MP Biomedicals) in line with to the manufacturer's instructions [16]. Further shotgun metagenomic library preparation was done by fragmenting the DNA at 300 bp (Covaris) and following the manual of the Ion Plus Fragment Library kit (ThermoFisher Scientific, USA). That included the following sample processing steps: (1) end repair after the physical fragmentation and clean-up with NucleoMag magnetic beads (Macherey-Nagel, Düren, Germany), (2) adaptor ligation, nick-repair, and clean-up, (3) size selection in the range 360–440 bp, performed with BluePippin DNA 2% Dye-Free Agarose gel cassette with V1 Marker, and clean-up, and (4) amplification and clean-up. Samples were sequenced using Ion Proton sequencer with Ion PI Chip Kit v3 (>3000000 reads/sample) [17, 18].

### Sequence analysis and statistics

Raw data from the sequencer were processed as follows: adapters were removed with cutadapt 1.16, sequences were trimmed with Trimmomatic v0.38 (5bp window, quality threshold = 20, average quality = 20, minimal length = 75), mapping was performed with bowtie2-2.3.5.1 using Homo sapiens genome Ensembl GRCh38 release-90 reference to remove host DNA sequences. Information on read numbers during sequence preprocessing has been summarized in S3 Table.

Composition and functionality from the remaining sequences of gut microbiome samples were analyzed using the HUMAnN2 pipeline [19], and taxonomic data were obtained with MetaPhlAn2 [20], analyses were performed with default parameters. Species level alpha diversity was calculated as the exponential of the Shannon index resulting in the effective number of species, and beta diversity was analyzed with non-metric multidimensional scaling (NMDS) using Bray-Curtis distances. Results of beta diversity were compared between subgroups with permutational multivariate analysis of variance—PERMANOVA. To explain the effects of environmental variables, adonis function (vegan package) was used to test the significance of individual variables, and complemented with Canonical Correspondence Analysis (CCA) and visualized with biplot using R software (version 3.6.0) [21]. Evaluation of variables of interest was performed in two cases: (1) for all samples–both groups, baseline and follow up–to evaluate the contribution of age, gender and BMI; (2) only for T2D patient samples–to evaluate possible effect of the different prescribed metformin doses. Changes during metformin therapy and differences between study subgroups within the taxonomic and functional profiles were evaluated by R package *limma* using *voom* transformation with sample-specific quality weights (further referred as limma+voom). All tests were adjusted by age, gender, and BMI, false discovery rate (FDR) adjusted values were used. T2D group data were adjusted by baseline HbA_1c_ levels. Only taxa present in ≥10% of samples were included. To compare metformin therapy response groups, the corrected data matrix was used for sparse Partial least squares discriminant analysis (sPLS-DA), a supervised model to reveal microbiota variation between groups. Key taxonomic groups responsible for the differential microbiota structure were detected using the “splsda” function in the R package “mix Omics” [22], tuning of sPLS-DA parameters was performed to determine the main taxonomic groups that enable discrimination of the subgroups with the lowest possible error rate. Taxonomic groups with variable importance in projection (VIP) > 1.5 were considered to be important contributors to the model. Additional cellular function enrichment analysis and visualization of functional profile data were performed using the Omics Dashboard integrated into MetaCyc. The dashboard computes enrichment p-values using Grossmann's parent-child-union variation of the Fisher-exact test (applying the FDR multiple hypothesis correction) and then transforms each p-value to an enrichment score: -log10 (p-value). Significance threshold <0.05 [23]. Statistical significance for changes/differences of the Shannon index and other analyzed parameters was evaluated by the Wilcoxon signed-rank test. Data normalizations were performed as integrated into the used tools, paired comparisons were used when appropriate.

### Validation cohort

To validate the results of the performed sPLS-DA analysis, we included another independent cohort of 58 newly diagnosed T2D patients. Inclusion/exclusion criteria, sample collection guidelines and design were the same as for the OPTIMED cohort, however, data were obtained from a different sequencing platform. Sequencing data preprocessing and statistical analysis were performed as described for OPTIMED cohort. Detailed information on methods for sample and data processing of Validation cohort is provided in S2 Text.

## Results

### Characterization of study cohorts

In total 100 samples were collected and analyzed from the OPTIMED cohort, and 103 samples from the healthy individuals. The characterization of the analyzed groups is summarized in Table 1. The average ±SD sequencing depth was 4.6 M ± 2.4 M raw reads per sample. During the clinical trial, two healthy participants withdrew from the study prematurely due to GI-SE, therefore no data on M7d time point were available for these two participants.

**Table 1:** Characteristics of the analyzed cohorts.

*Characteristic*	*Healthy individuals*, *N = 35*	*T2D patients*, *N = 50*	*Validation cohort (T2D patients)*, *N = 58*
Males/females, n (%)	10 (28.6%) / 25 (71.4%)	22 (44%) / 28 (56%)	30 (51.7%) / 28 (28.3%)
Age (years), mean ± SD	31.5 ± 10.2	58.6 ±12.5	58.2 ± 10.3
BMI, mean ± SD	24.5 ± 3.2	34.8 ± 6.7	34.0 ± 5.9
HbA_1c_ (mmol/mol) mean ± SD	32.2 ± 1.8	66.1 ± 0.5	59.6 ± 0.5
HbA_1c_ (%), mean ± SD	5.1 ± 0.5	8.2 ± 2.1	7.6 ± 2.0
Creatinine (μmol/l), mean ± SD	67.6 ± 11.6	68.6 ± 13.7	67.3 ± 17.0
ALAT (U.l), mean ± SD	23.5 ± 10.7	40.3 ± 21.8	46.0 ± 30.7
TG (mmol/l), mean ± SD	1.3 ± 1.0	2.4 ±1.8	2.4 ± 1.6
HDL-C (mmol/l), mean ± SD	1.6 ± 0.4	1.2 ± 0.3	1.3 ± 0.4
LDL-C (mmol/l), mean ± SD	2.8 ± 0.8	3.6 ± 1.0	3.4 ± 1.3
Metformin dose (mg/day), mean ± SD	1700 ± 0	1146 ± 702	1006 ± 455

T2D - type 2 diabetes; SD–standard deviation; BMI–body mass index; ALAT—alanine transaminase, TG – triglycerides, HDL-C–High-density lipoprotein cholesterol, LDL-C – Low-density lipoprotein cholesterol.

Retrospective analyses of the questionnaire data from the OPTIMED cohort revealed that few patients had some deviations from the expected study design: one patient was assigned to diet change in the first week, without any drug treatment; another used sulfonylurea group medication. These participants were excluded from the study group, leaving 48 T2D patients for further analysis.

**Fig 2:**
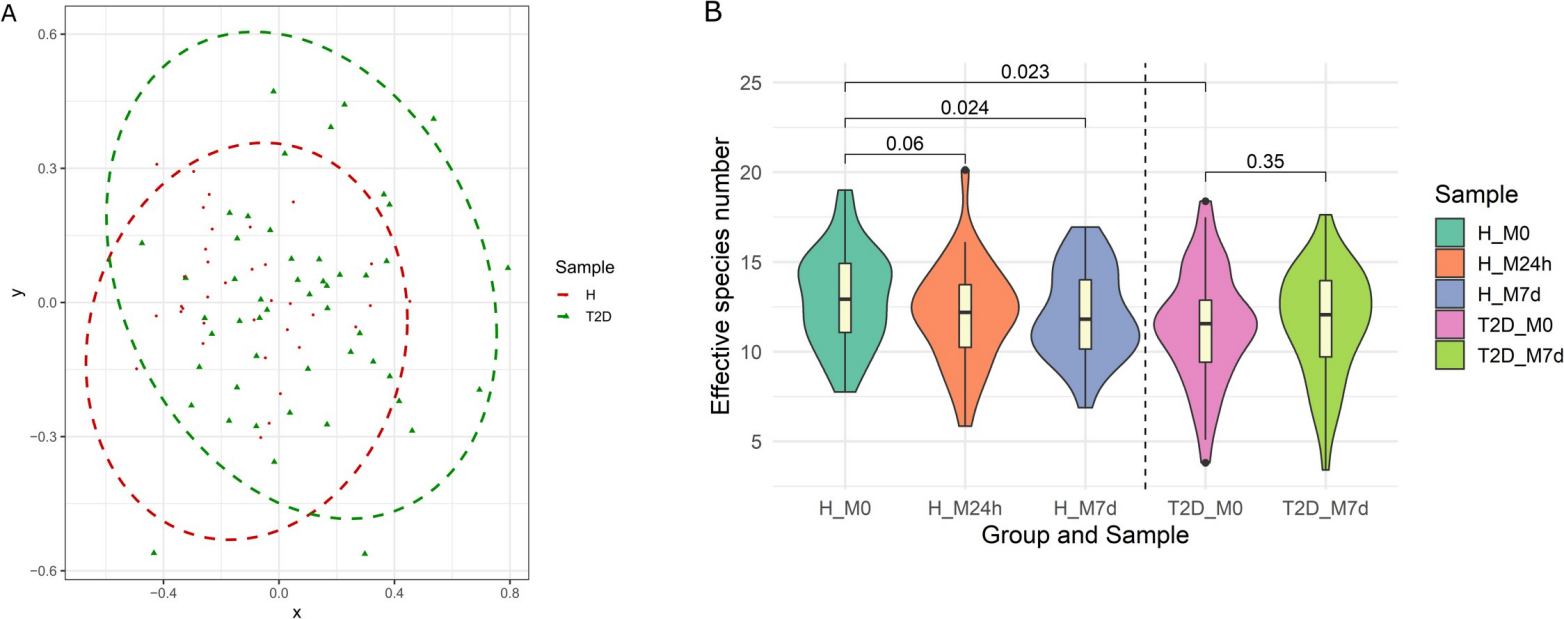
Diversity characteristics of analyzed samples. (A) Beta diversity characterizing and comparing samples before metformin therapy between healthy individuals (H) and OPTIMED cohort patients (T2D). Ellipses represent the 95% confidence interval surrounding each group of samples. Different symbols represent the participants of the study. Red circles correspond to healthy individuals while green triangles represent type 2 diabetes patients. (B) Alpha diversity calculated in all analyzed time points. Groups marked as follows: H–healthy individuals; T2D – type 2 diabetes patients. Samples: M0—before starting metformin treatment; M24h – 24 hours after the first intake of metformin; M7d –after 7 days treatment with metformin. Violin plot representing the effective number of species combines boxplots, depicting the median value and interquartile ranges, with Kernel density plots.

When evaluating sample composition between both analyzed cohorts at baseline (M0 time point), it was possible to distinguish these groups based on beta diversity (Fig 2A). Differences were statistically significant (PERMANOVA: R^2^ = 0.035, p = 0.0015). Moreover, alpha diversity (Fig 2B) was significantly higher in the healthy cohort. To characterize the possible effect of available covariates and their contribution to the variation in the taxonomical composition, CCA was performed on all collected samples. We found the corresponding contribution of the analyzed cofactors: age (1.5%, p = 0.001), BMI (4.1%, p = 0.001), gender (1.4%, p = 0.001).

### Metformin-induced changes in the taxonomic profile

Metformin induced a significant decrease in effective species number in healthy individuals (M0 vs M7d –median M0 = 12.9, median M7d = 11.8; p = 0.024), supporting the results from our pilot study in a smaller group [11]. In T2D patients we observed a slight increase in the effective species number at the same time points (M0 vs M7d –median M0 = 15.6, median M7d = 12.1; p = 0.35) but this change was not significant (Fig 2B).

**Fig 3:**
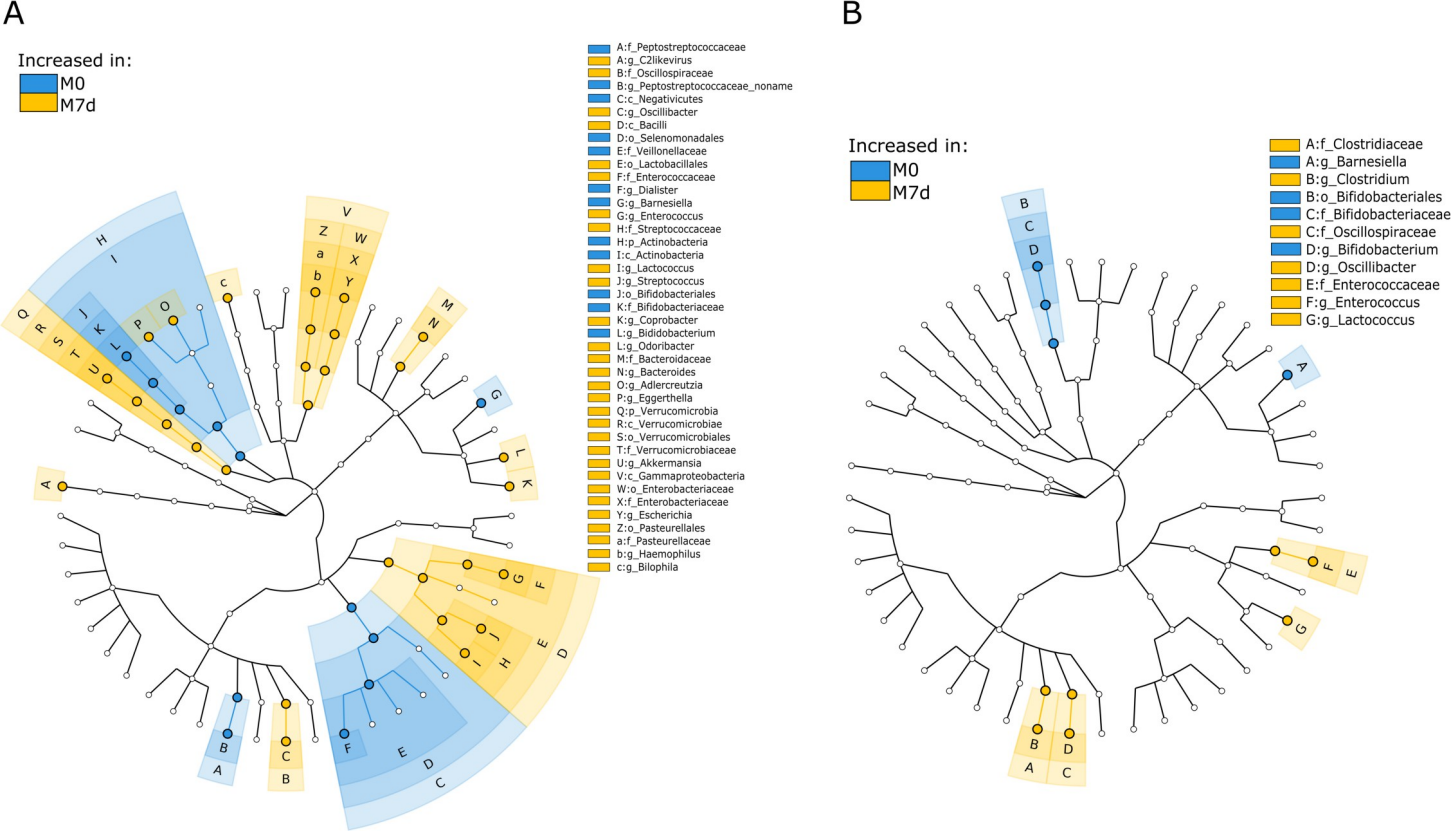
GraPhlAn cladogram for taxonomic composition changes during a week-long metformin administration. (A) healthy individuals, (B) type 2 diabetes patients. Samples are marked as follows: M0—before starting metformin treatment (blue); M7d –after 7 days treatment with metformin (yellow). Colors of nodes and shading indicate the microbial lineages that are enriched within corresponding samples. Only differentially abundant taxa at the genus or higher taxonomic ranks are indicated. For detailed results in lower taxonomical levels, see S1 and S2 Figs.

After a weeklong metformin treatment, the healthy group showed 115 significantly changed features at various taxonomic levels, and the OPTIMED cohort showed 26 changed features (Fig 3, S1 and S2 Figs, S2 Table). At species level, only four alterations overlapped between both study groups–a decrease in the abundance of *Clostridium bartlettii* and *Barnesiella intestinihominis*, and an increase in the abundance of *Parabacteroides distasonis* and *Oscillibacter* unclassified–while other changes in the taxonomic profile were specific to the analyzed cohorts.

To ensure the accuracy of the results, we additionally tested the possible effects of different metformin doses. When analyzed by CCA, the dose of metformin was not a significant co-factor in influencing the microbiome composition (0.9%, p = 0.56) in the patient cohort.

### Taxonomic differences associated with the treatment side effects and efficacy

**Fig 4:**
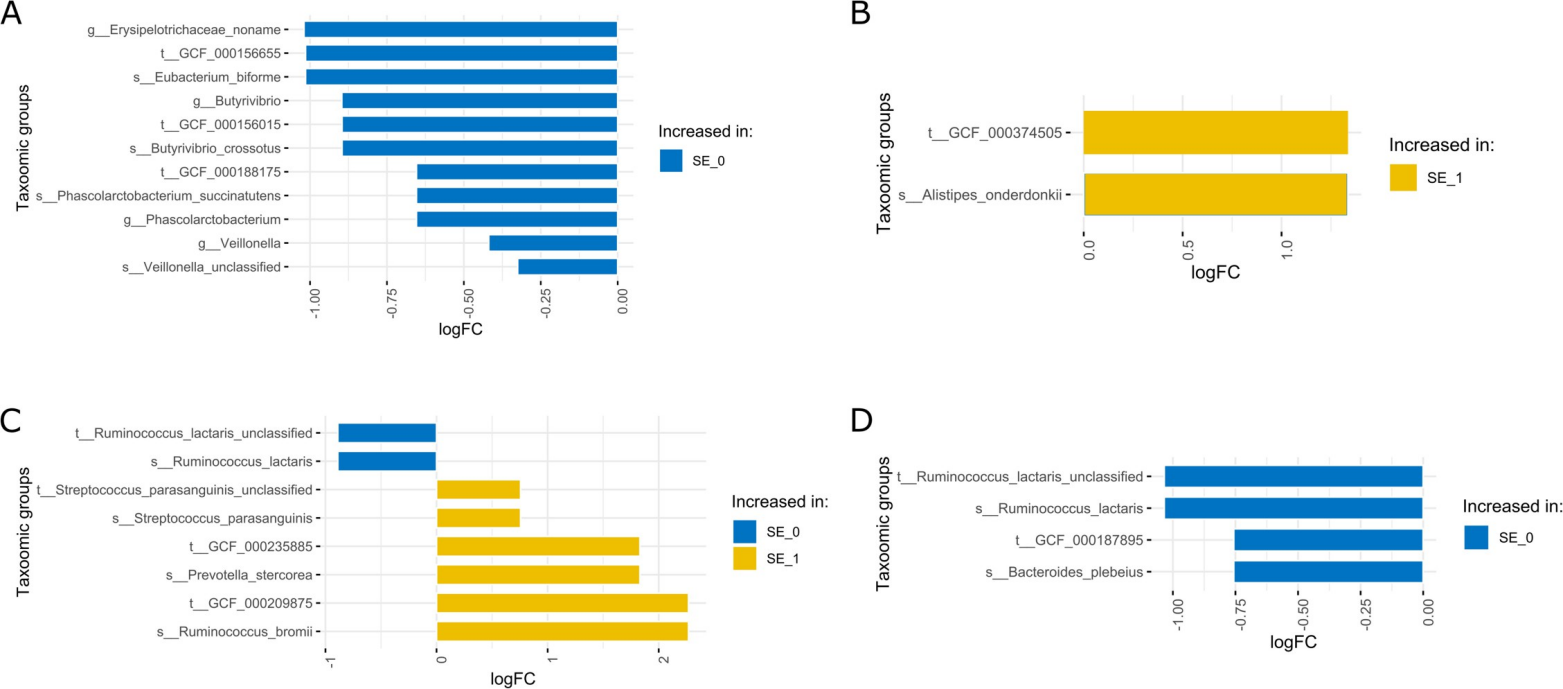
Differences in gut microbiome taxonomic composition between patients with severe side effects (SE_1) and no or mild reported side effects (SE_0) in both cohorts at all analyzed time points. (A) Healthy individuals at M0, (B) healthy individuals at M7d, (C) OPTIMED cohort at M0, (D) OPTIMED cohort at M7d. Taxa enriched in patients with severe side effects are indicated with yellow color, and taxa enriched in patients with no or mild side effects are in blue. Samples: M0 – before starting metformin treatment; M7d –after 7 days treatment with metformin.

During the study, all observed SE were registered in the study group-specific questionnaires. Study subjects from each cohort were divided into two groups according to the type of registered SE during the usage of metformin. The first group included participants with no or mild SE defined by headache, meteorism (tympanites), stomach ache, nausea, and loss of appetite; and the second group included individuals with severe SE defined by loose stools 1–3 times a day, diarrhea, vomiting. In the OPTIMED cohort, nine individuals had severe SE and 39 did not report any SE while in the group of healthy individuals 21 participants had mild or no SE and 14 had severe intolerance. For a detailed analysis of possible microbiome mediated mechanisms and predictors of metformin-induced GI-SE, we performed a comparison of taxonomic profiles between these defined groups at the analyzed study time points (Fig 4).

As a next step, we evaluated the association between the presence of specific taxonomic groups before therapy start (M0) and the efficacy of metformin therapy (changes in HbA_1c_ levels during the first three months of therapy) in the cohort of T2D. Two of the patients had withdrawn from the OPTIMED study before the three-month time point (M3m), therefore, they were excluded from this analysis. We divided the remaining OPTIMED cohort (N = 46) into two groups characterized in Table 2. Metformin's therapeutic effects induced a statistically significant reduction in HbA_1c_ levels during the first three months of therapy in both groups (Responders (p = 0.0002), Non-responders (p = 0.001)), but not on BMI. For any further analyses, we performed a correction by baseline HbA_1c_ value.

**Table 2:** Characteristics of OPTIMED cohort’s subgroups divided by response to metformin therapy during the first three months of therapy.

*Characteristic*	*Responders*, *N = 18*	*Non-responders*, *N = 28*	*p-value*
Males/females, n (%)	12 (66.7%) / 6 (33.3%)	8 (28.6%) / 20 (71.4%)	-
Age (years), mean ± SD	53.6 ± 10.5	61.3 ± 12.5	0.02
M0 BMI, mean ± SD	35.8 ± 7.8	34.6 ± 5.4	0.50
M3m BMI, mean ± SD	35.1 ± 7.2	34.0 ± 5.8	0.77
M0 HbA_1c_ (mmol/mol), mean ± SD	83.6 ± 4.9	50.8 ± 12.6	2.36 E-7
M0 HbA_1c_ (%), mean ± SD	9.8 ± 1.7	6.8 ± 1.0	2.36 E-7
M3m HbA_1c_ (mmol/mol), mean ± SD	53.0 ± 13.7	48.6 ± 12.6	0.04
M3m HbA_1c_ (%), mean ± SD	7.0 ± 0.9	6.6 ± 1.0	0.04

SD–standard deviation; BMI–body mass index; M0 –before starting metformin treatment; M3m –after three months of metformin treatment.

Firstly, we tested for differentially abundant taxonomic groups between both OPTIMED subgroups. When comparing taxonomic profiles we observed an increased abundance of species *Prevotella copri* in Non-Responders group (logFC = -2.8, FDR = 0.01) at M0 time point. No significant differences in the effective species number were detected when comparing these subgroups.

**Fig 5:**
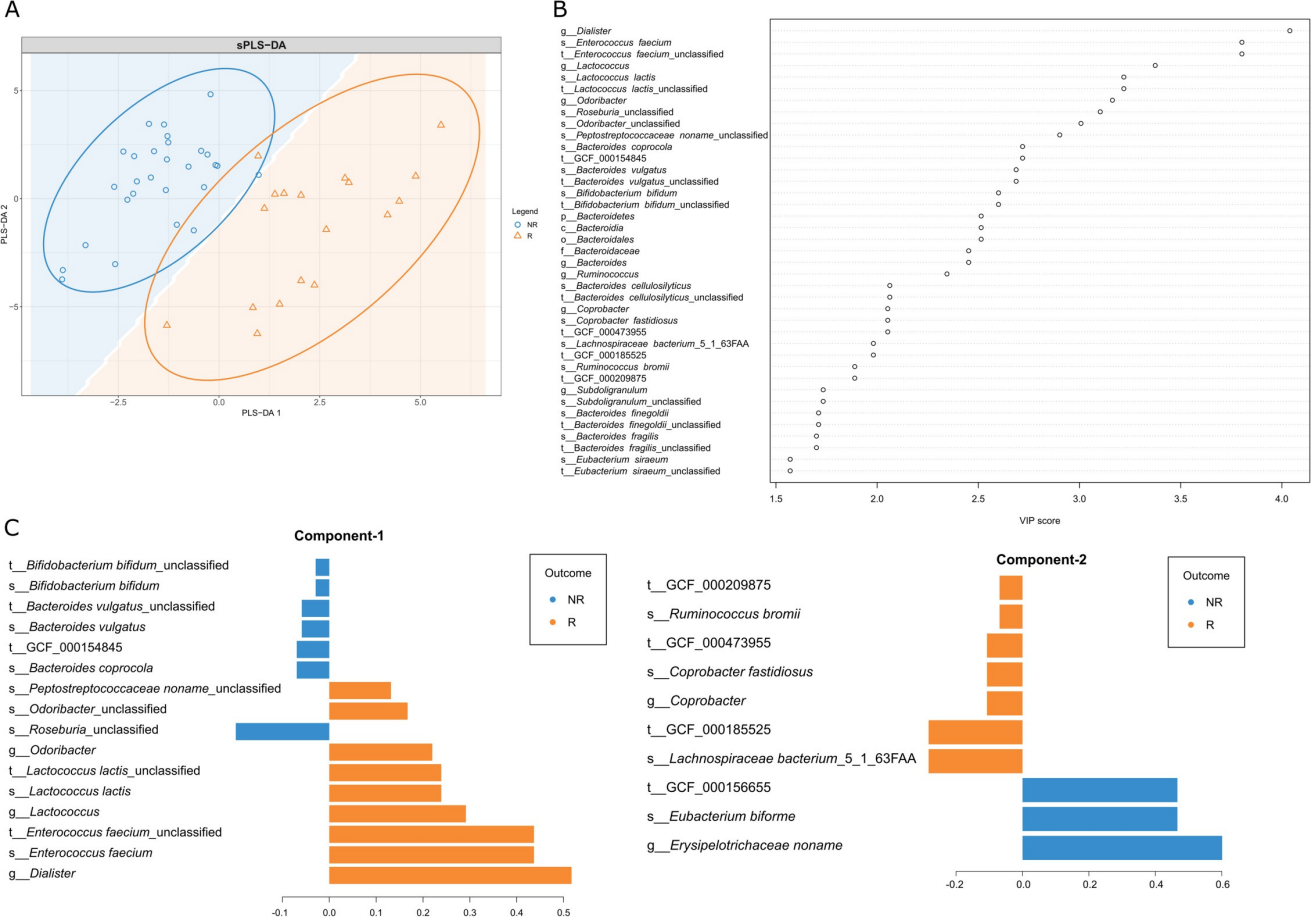
Sparse Partial Least Squares Discriminant Analysis (sPLS-DA) of OPTIMED cohort subgroups at M0 time point. (A) Sample plot depicting the first two sPLS-DA components with 95% confidence level ellipse plots. The background coloring describes the predicted area for each class, defined as the 2D surface where all points are predicted to be of the same class. Samples and subgroups coded as follows: R–Responders, orange triangles; NR–Non-Responders, blue circles. (B) VIP (variable importance projection) score dot-chart classified by sPLS-DA. Depicted taxonomic groups with VIP≥1.5 in the first component. (C) The contribution of each taxonomic group on the first and second components, the length of the bar represents the importance of each feature to the component (importance from bottom to top). Colors indicate the patient subgroup (NR (blue) vs R (orange)) in which the taxonomic group is most abundant.

Secondly, we performed an additional sPLS-DA model to explore which taxonomic groups could discriminate patients belonging to one of the defined subgroups, and the VIP score was used to assess the contribution of each analyzed taxonomic unit (Fig 5). In total 43 taxonomic groups were detected with VIP score >1.5 (the full list is summarized in S1 Table).

Finally, we performed the same analysis on the independent validation cohort (characteristics summarized in Table 1). In result, for sPLS-DA model six taxonomic units overlapped: species *Bacteroides vulgatus* and its strain *Bacteroides vulgatus* unclassified, genus *Erysipelotrichaceae* noname and its species *Eubacterium biforme*, and its strain GCF 000156655, and species *Ruminococcus obeum*.

### Functional analysis

**Fig 6:**
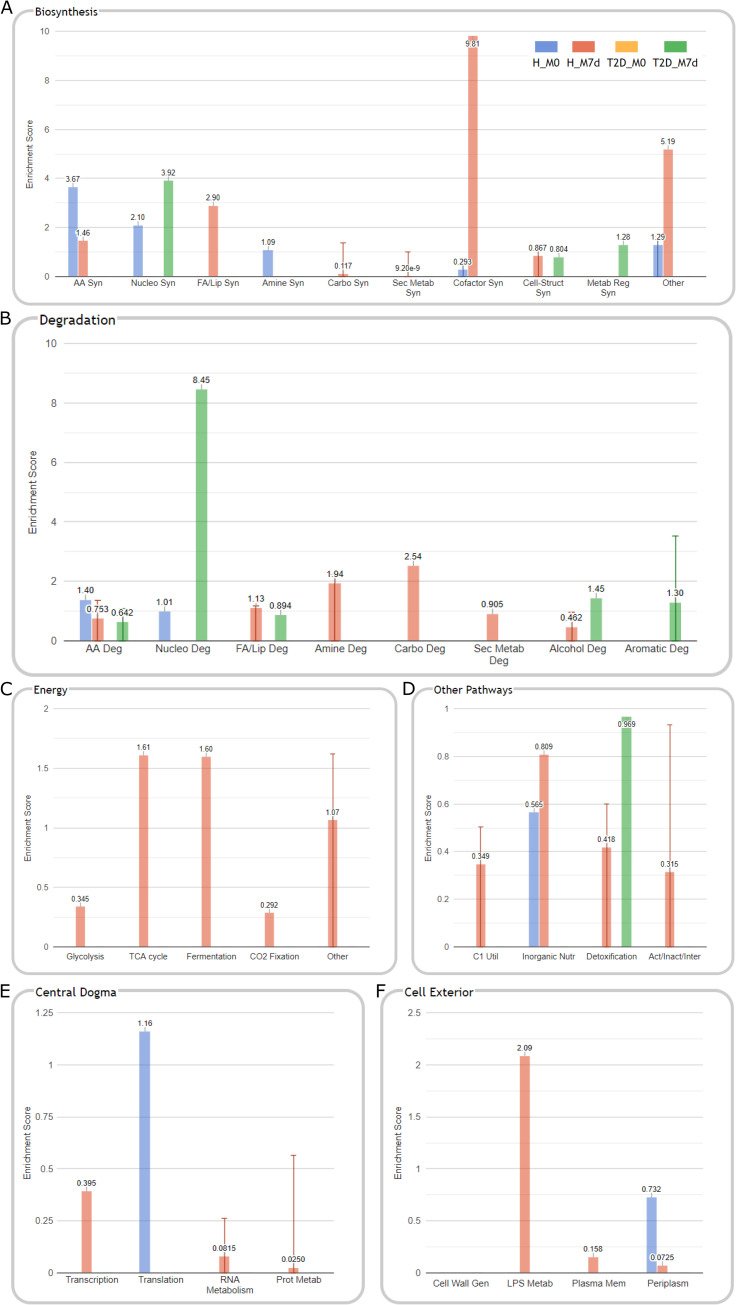
Cellular function enrichment analysis comparing functional profiles before and after 7 days long metformin therapy both in the OPTIMED cohort and in healthy individuals. Respectively, H_M0 (blue) and T2D_M0 (yellow) results represent data on pathways and functions reduced during metformin therapy and H_M7d (red) and T2D_M7d (green) – pathways and functions increased, only significant (p<0.05) enrichment categories are shown. Enrichment results are depicted in the following set of functional panels: (A) Biosynthesis, (B) Degradation, (C) Energy, (D) Other pathways, (E) Central dogma, (F) Cell exterior. Groups marked as follows: H–healthy individuals; T2D – type 2 diabetes patients. Samples: M0—before starting metformin treatment; M7d –after 7 days treatment with metformin. Pathway abbreviations: (A) AA Syn – amino acid biosynthesis; Nucleo Syn – nucleoside and nucleotide biosynthesis; FA/Lip Syn–fatty acid and lipid biosynthesis; Amine Syn–amine and polyamine biosynthesis; Carbo Syn – carbohydrate biosynthesis; Sec Metab Syn–secondary metabolite biosynthesis; Cofactor Syn–cofactor, carrier, and vitamin biosynthesis; Cell-Struct Syn–cell structure biosynthesis; Metab Reg Syn–metabolic regulator biosynthesis. (B) AA Deg–amino acid degradation; Nucleo Deg–nucleoside and nucleotide degradation; FA/Lip Deg–fatty acid and lipid degradation; Amine Deg–amine and polyamine degradation; Carbo Deg –carbohydrates and carboxylates degradation; Sec Metab Deg–secondary metabolite degradation; Alcochol Deg–alcohol degradation; Aromatic Deg–aromatic compound degradation. (D) C1 Util–C1 compound utilization and assimilation; Inorganic Nutr – inorganic nutrient metabolism; Act/Inact/Inter—Activation/Inactivation/Interconversion. (E) Prot Metab–protein metabolism. (F) Cell Wall Gen–cell wall biogenesis/organization proteins; LPS Metab – Lipopolysaccharide Metabolism Proteins; Plasma Mem–plasma membrane proteins; Periplasm–periplasmic proteins.

Using the advantage of shotgun metagenomics data, we further evaluated the changes in possible functions of the analyzed gut microbiomes. This task was performed by analyzing the differential abundance of signaling pathways calculated by HUMAnN2 (proportional to the number of complete "copies" of the pathway in the community) within both studied cohorts. As a result, we identified 24 significant features in the OPTIMED cohort and 118 features in the healthy group (the specific pathways depicted in S3 and S4 Figs) with changed abundance during the metformin therapy. To gain a detailed insight of general biological meaning represented by these functional changes we performed a cellular function enrichment analysis (Fig 6). Enrichment scores were calculated from logFC and adjusted p-values obtained from differential pathway abundance analysis comparing M7d versus M0 samples both in healthy and in T2D cohorts.

To perform targeted evaluation of some specific metabolic functions previously described to be associated with metformin, we took a detailed look under some of the enrichment categories. Firstly, under Cell Structure Biosynthesis category, increased peptidoglycan biosynthesis was observed in both analyzed cohorts, and increment in pathways dedicated to biosynthesis of LPS precursor Lipid IVa was detected only in the healthy cohort.

Secondly, after detailed analysis of enriched pathways under Cofactor, Carrier, and Vitamin Biosynthesis (Fig 6), in vitamin biosynthesis subcategory we observed changes only in the healthy cohort–reduced folate biosynthesis, increased thiamine and vitamin B6 biosynthesis.

## Discussion

Our study has added new data on several most likely universal metformin effects on the human gut microbiome profile and presents novel data for therapeutic efficacy and tolerance prediction in newly diagnosed T2D patients. Also, we have characterized the differences representing metformin effects in T2D patients and healthy individuals, accenting the need for additional microbiome studies in groups with different responses to metformin therapy, both, in context of geographical localization and metformin targets outside the T2D.

The main strengths of our study are the longitudinal design examining the short-term metformin therapy effects on well-characterized treatment naïve patients and the additional study of healthy individuals receiving metformin. The used methodology of shotgun metagenome sequencing also improves the quality of study allowing discussing species-level data and changes in the functional profile. The main limitation is the relatively small sizes of study groups, however, we are the first to present short-term metformin effects observed after a weeklong therapy in newly diagnosed patients and it should be noted that the previously published longitudinal studies are similarly sized or even smaller [9, 10]. We also did not include a placebo arm and blinded design that could uncover possible metformin independent effects. In addition, the higher metformin dose given to the participants of the healthy cohort is a confounding limitation for comparison of both analyzed groups. Long-term follow-up for our study groups would also provide additional opportunity to evaluate the stability of observed effects, however, specifically, the short-term therapy results are with high clinical significance as it is known that the highest incidence of SE is observed during the first weeks [24].

In both healthy individuals and newly diagnosed T2D patients, we observed a metformin-induced reduction in the abundance of *Clostridium bartlettii* (also called *Intestinibacter bartlettii*–latest classification [25]). The role of this species is still unclear as its abundance has shown a negative correlation with markers for insulin resistance [26], but in other studies, it has been described as a robust biomarker for Crohn’s disease and ulcerative colitis [27]. Importantly, a reduced abundance of *Intestinibacter* genus has been observed in previous metformin studies [8, 9, 11], thus, suggesting it to be one of the universal markers characterizing metformin effects on the gut microbiome.

One of the most intriguing findings was the increased abundance of *Parabacteroides distasonis* species. This taxonomic group has been recently associated with improved insulin sensitivity in obese human subjects [28], alleviated obesity and metabolic dysfunctions in mice [29], and has been proven to negatively correlate with fasting blood glucose levels [30]. In addition, some recent studies of metformin effects have observed an increase in the abundance of this species or obtained associations with therapy outcomes [31, 32].

At genus level, as possibly negative metformin effect, decrement in *Bifidobacterium* was observed in both analyzed cohorts. This taxon plays an important role in human health maintenance and is widely used as probiotics, as well as its reduction can be used as a biomarker for certain diseases [33]. Co-administration of *Bifidobacterium bifidum* G9-1 with metformin even has shown beneficial effects on reducing GI-SE [34]. However, the observed reduction in this genus needs more research, as effects on health are most likely species and strain specific, and our result is in contradiction with results of previous studies, for example, previously it has been reported that metformin enhances growth of species *Bifidobacterium adolescentis* in pure cultures [9].

We confirmed the previously observed increase in the abundance of *Escherichia coli* [9, 11] only in the group of healthy individuals. In this study cohort, we observed the highest incidence of SE, therefore, contributing the hypothesis of these changes as a possible basis for metformin intolerance. Interestingly, in previous studies, the highest SE occurrences (up to 50% and higher) have been observed during metformin administration in non-diabetic cohorts, such as healthy volunteers [10, 11] or polycystic ovary syndrome patients [35] compared to T2D. Regarding the possible beneficial effects of metformin specific to the healthy cohort, we found a metformin-induced reduction in *Dialister invisus* and *Bifidobacterium longum*, both associated with intestinal permeability and compromised gut health [36].

In the search for possible microbiome signatures describing or predicting the therapy tolerance, we observed different profiles in both analyzed cohorts. One of the taxa specific to the subgroup with severe SE in OPTIMED cohort (M0 time point) was *Streptococcus parasanguinis*, previously shown to be increased in individuals who use platelet aggregation inhibitors and proton pump inhibitors [37], which both are groups of medications frequently prescribed for the treatment of diabetes comorbidities. This result could indicate the possible effects of polypharmacy on the metformin’s interaction with gut microbiome and the subsequent therapy tolerance. In contrast, species *Ruminococcus lactaris* which was enriched in OPTIMED subgroup with no or mild SE both at M0 and M7d samples, has been enriched in healthy individuals compared to T2D patients or obese subjects in various populations [38, 39], as well as negatively associated with statin use [40]. Within the healthy cohort (subgroup with no or mild SE) both before and after metformin use was characterized by an increased abundance of *Phascolarctobacterium succinatutens*, a succinate-consumer and substantial producer of short-chain fatty acids acetate and propionate. Therefore, this taxon has been associated with the metabolic state and even the mood of the host [41]. Some of our observed taxa have been previously characterized as discriminants for SE development in a healthy cohort, such as *Alistipes* [10]. Altogether, these results give new insights into possible microbiome signatures for future prediction of therapy tolerance, but additional studies in larger patient cohorts with a well-characterized incidence of SE are needed to confirm our data.

Taking into account the widely known variation of metformin’s therapeutic efficacy [42] we used the advantage of our longitudinal and well described OPTIMED cohort to search for potential microbiome-based markers as predictors. Firstly, we compared the taxonomic profiles in the subgroups crossectionally–at M0 time point before starting metformin therapy–with a fitted linear model for a series of arrays. The main finding in this step was the increment in abundance of *Prevotella copri* in the samples from Non-Responders to the therapy. Previous studies describing the possible functionality of *P*.*copri* species present contradictory data. Research results suggest both *P*.*copri* mediated beneficial effects on the host’s metabolic profile as a succinate producer [43] and its induced increase in insulin resistance, glucose intolerance, and lipopolysaccharides plasma levels [44, 45]. However, the latest data indicate that strain-specific effects most likely explain this controversy, and the strain-level composition might be diet dependent [46]. Thus for the future development of biomarker-based approaches, strain-level data should be analyzed to account for the population and lifestyle specific microbiome composition with an aim to precisely predict its dependent functionality.

Secondly, the performed sPLS-DA analysis revealed a broad list of key taxa discriminating both therapy response subgroups at M0 time point. More precisely, the microbiome of the Responders group at baseline was enriched with various taxonomic groups characterized as potentially probiotic. For example (1) *Enterococcus faecium* significantly decreased body weight, serum lipid levels, blood glucose level, and insulin resistance in rats fed with a high-fat diet [47]; (2) several *Lactococcus lactis* strains have shown the ability to reduce hyperglycemia, improve glucose tolerance and insulin secretion [48, 49]; (3) bacteria from *Odoribacter* genus have been associated with a healthy fasting serum lipid profile [26], and displayed a negative correlation with insulin resistance [50]. The top result from this analysis–genus *Dialister* – has been characterized as a taxon possibly mediating the beneficial effects on the metabolic profile of whole-grains [51], however more data on underlying species are needed. In contrast, the various species from the predominant genus *Bacteroides*, found to be specific to the group of Non-responders, has been previously described in higher abundance in type 1 and T2D patients [52, 53], as well as associated with a negative impact on metabolic health [44]. Nevertheless, it is important to accent that the possible biological role of this genus is highly variable due to numerous species and strains within it, therefore, the interaction with the host can be both beneficial and harmful [54] and need to be further studied in the context of metformin response.

Data validation of sPLS-DA model in the independent cohort highlighted six taxonomic groups from three phylogenetic branches that have been previously associated with T2D, glucose tolerance, insulin resistance, and blood glucose levels, however, the results are highly conflicting and mostly population specific [45, 55–57]. These results highlight the urgent need for further population specific clinical studies to develop highly precise microbiome-based prediction tools for therapy efficacy.

To our best knowledge, we are the first to report the results of such analysis combining metformin therapy efficacy and microbiome profile data from newly diagnosed and treatment naïve T2D patients. Most importantly, comparing to studies with a similar design that compare Responders and Non-Responders to the antidiabetic therapy but analyze other targets, we have performed data correction by baseline HbA_1c_ measurement, to reduce biases created by frequently observed higher baseline values in the Responders group, as suggested previously [58]. Nevertheless, it is important to point out that other therapy efficacy influencing indicators should be evaluated to more precisely distinguish microbiome-related effects from e.g. presence of genetic factors previously associated with efficacy.

As for the functional profile, only a portion of the observed results has been previously characterized. A large number of the significantly changed pathways and subsequently the results of enrichment analysis during metformin use were representing the increment in pathways characterizing synthesis of lipopolysaccharides and peptidoglycans (under *Cell-Struct Syn* in Fig 6), which is another signature of metformin effects [9, 59]. These changes were mainly found in the healthy cohort, most likely accounting for the high number of observed fluctuations in the taxonomical profiles. For example, a recent study employing genome-scale metabolic modelling has shown that lipopolysaccharide synthesis, nucleotide sugar metabolism, and amino acid metabolism (under *Cell-Struct Syn*, *Nucleo Syn*, *Nucleo Deg*, *AA Syn*, *AA deg* in Fig 6) are pathways most likely effected by abundance changes in such taxa as *Escherichia* spp. and *A*. *muciniphila* [60]. Compared to other analyses of metformin-induced changes in the functional profile performed in T2D cohorts, we observed similar changes, such as an increase in lysine and threonine degradation (in healthy cohort), and sugar nucleotide biosynthesis (in OPTIMED cohort) [9]. Interestingly, the enriched cellular functions appeared to be cohort-specific and in cases when similar functional changes are observed, the observed underlying mechanisms differed.

In addition, as metformin is known to be associated with vitamins B level alterations and even deficiencies [61, 62], we as well observed metformin induced changes in various vitamin B pathways, however, only in the healthy cohort. The inhibition of folate metabolism have been characterized as one of the mechanisms behind metformin effects on increased lifespan in *C*.*elegans* [63]. However, the suppression of folate producing bacteria has been proposed as one of the causes for GI-SE [64], therefore, indicating a possible explanation for the high prevalence of SE observed specifically in the healthy cohort.

The large disparity in the observed microbiome profile changes in both cohorts could be explained by initial differences (as depicted in Fig 2). Moreover, as the T2D cohort is expected to be more heterogeneous [65], it explains the smaller number of significantly changed features in OPTIMED patients. In addition, our results have approved some seemingly universal microbiome signatures for metformin therapy and displayed new data on microbiome changes, most likely responsible for population-specific effects dependent on health status as well as geographical localization. Our results on the prediction of therapy tolerance and efficacy may reveal novel biomarkers, which need to be further studied to fully characterize the strain-level dynamics and validated in larger cohorts. These results highlight the need to develop personalized medicine based approaches based on gut microbiome testing before starting the therapy and will serve as the basis for further studies on microbiome modulation techniques to improve both metformin therapeutic efficacy and tolerance and, therefore, the quality of life of patients.

## Supporting information

S1 Text List of inclusion/exclusion criteria. (DOCX)

S2 Text Methodological description of validation cohort sample and data processing. (DOCX)

S1 Fig Metformin induced differences in gut microbiome taxonomic composition in T2D cohort. Differentiating feature analysis was carried out with limma+voom, adjusted p-value cut-off = 0.05. Samples coded as follows: M0 –before starting metformin treatment (blue, negative logFC), and M7d – 7 days after the first intake of metformin (yellow, positive logFC). logFC–log fold change.
(TIF)

S2 Fig Metformin induced differences in gut microbiome taxonomic composition in healthy cohort. Differentiating feature analysis was carried out with limma+voom, adjusted p-value cut-off = 0.05. Samples coded as follows: M0 –before starting metformin treatment (blue, negative logFC), and M7d – 7 days after the first intake of metformin (yellow, positive logFC). logFC–log fold change.
(TIF)

S3 Fig Differences in abundance of signaling pathways induced by metformin therapy in T2D patient cohort. Differentiating feature analysis was carried out with limma+voom, adjusted p-value cut off = 0.05. Samples coded as follows: M7d – 7 days after the first intake of metformin (blue, positive logFC). logFC–log fold change.
(TIF)

S4 Fig Differences in abundance of signaling pathways induced by metformin therapy in healthy individuals. Differentiating feature analysis was carried out with limma+voom, adjusted p-value cut off = 0.05. Samples coded as follows: M0 –before starting metformin treatment (blue, negative logFC), and M7d – 7 days after the first intake of metformin (yellow, positive logFC). logFC–log fold change.
(TIF)

S1 Table Taxonomic groups with VIP score >1.5 in at least one of the first two components from sPLS-DA analysis. (DOCX)

S2 Table Effect sizes and p-values of performed comparisons with limma+voom analysis. (XLSX)

S3 Table Sequence preprocessing information. (XLSX)

## References

[1] Skyler JS, Bakris GL, Bonifacio E, Darsow T, Eckel RH, Groop L, et al. Differentiation of Diabetes by Pathophysiology, Natural History, and Prognosis. Diabetes. 2017;66(2):241–55. doi: 10.2337/db16-0806 27980006 PMC5384660

[2] Zhou J, Massey S, Story D, Li L. Metformin: An Old Drug with New Applications. Int J Mol Sci. 2018;19(10). doi: 10.3390/ijms19102863 30241400 PMC6213209

[3] Kahn SE, Haffner SM, Heise MA, Herman WH, Holman RR, Jones NP, et al. Glycemic durability of rosiglitazone, metformin, or glyburide monotherapy. N Engl J Med. 2006;355(23):2427–43. doi: 10.1056/NEJMoa066224 17145742

[4] Knowler WC, Barrett-Connor E, Fowler SE, Hamman RF, Lachin JM, Walker EA, et al. Reduction in the incidence of type 2 diabetes with lifestyle intervention or metformin. N Engl J Med. 2002;346(6):393–403. doi: 10.1056/NEJMoa012512 11832527 PMC1370926

[5] Whang A, Nagpal R, Yadav H. Bi-directional drug-microbiome interactions of anti-diabetics. EBioMedicine. 2019;39:591–602. doi: 10.1016/j.ebiom.2018.11.046 30553752 PMC6354569

[6] Petrosino JF. The microbiome in precision medicine: the way forward. Genome Med. 2018;10(1):12. doi: 10.1186/s13073-018-0525-6 29471863 PMC5824491

[7] Karlsson FH, Tremaroli V, Nookaew I, Bergstrom G, Behre CJ, Fagerberg B, et al. Gut metagenome in European women with normal, impaired and diabetic glucose control. Nature. 2013;498(7452):99–103. doi: 10.1038/nature12198 23719380

[8] Forslund K, Hildebrand F, Nielsen T, Falony G, Le Chatelier E, Sunagawa S, et al. Disentangling type 2 diabetes and metformin treatment signatures in the human gut microbiota. Nature. 2015;528(7581):262–6. doi: 10.1038/nature15766 26633628 PMC4681099

[9] Wu H, Esteve E, Tremaroli V, Khan MT, Caesar R, Manneras-Holm L, et al. Metformin alters the gut microbiome of individuals with treatment-naive type 2 diabetes, contributing to the therapeutic effects of the drug. Nat Med. 2017. doi: 10.1038/nm.4345 28530702

[10] Bryrup T, Thomsen CW, Kern T, Allin KH, Brandslund I, Jorgensen NR, et al. Metformin-induced changes of the gut microbiota in healthy young men: results of a non-blinded, one-armed intervention study. Diabetologia. 2019;62(6):1024–35. doi: 10.1007/s00125-019-4848-7 30904939 PMC6509092

[11] Elbere I, Kalnina I, Silamikelis I, Konrade I, Zaharenko L, Sekace K, et al. Association of metformin administration with gut microbiome dysbiosis in healthy volunteers. PLoS One. 2018. doi: 10.1371/journal.pone.0204317 30261008 PMC6160085

[12] de la Cuesta-Zuluaga J, Mueller NT, Corrales-Agudelo V, Velasquez-Mejia EP, Carmona JA, Abad JM, et al. Metformin Is Associated With Higher Relative Abundance of Mucin-Degrading Akkermansia muciniphila and Several Short-Chain Fatty Acid-Producing Microbiota in the Gut. Diabetes Care. 2017;40(1):54–62. doi: 10.2337/dc16-1324 27999002

[13] Rovite V, Wolff-Sagi Y, Zaharenko L, Nikitina-Zake L, Grens E, Klovins J. Genome Database of the Latvian Population (LGDB): Design, Goals, and Primary Results. J Epidemiol. 2018. 10.2188/jea.JE20170079 PMC6048300 29576601

[14] Sherifali D, Nerenberg K, Pullenayegum E, Cheng JE, Gerstein HC. The effect of oral antidiabetic agents on A1C levels: a systematic review and meta-analysis. Diabetes Care. 2010;33(8):1859–64. doi: 10.2337/dc09-1727 20484130 PMC2909079

[15] Kashi Z, Mahrooz A, Kianmehr A, Alizadeh A. The Role of Metformin Response in Lipid Metabolism in Patients with Recent-Onset Type 2 Diabetes: HbA1c Level as a Criterion for Designating Patients as Responders or Nonresponders to Metformin. PLoS One. 2016;11(3):e0151543. doi: 10.1371/journal.pone.0151543 26978661 PMC4792461

[16] Burbach K, Seifert J, Pieper DH, Camarinha-Silva A. Evaluation of DNA extraction kits and phylogenetic diversity of the porcine gastrointestinal tract based on Illumina sequencing of two hypervariable regions. Microbiologyopen. 2016;5(1):70–82. doi: 10.1002/mbo3.312 26541370 PMC4767427

[17] Janes VA, van der Laan JS, Matamoros S, Mende DR, de Jong MD, Schultsz C. Thermus thermophilus DNA can be used as internal control for process monitoring of clinical metagenomic next-generation sequencing of urine samples. J Microbiol Methods. 2020;176:106005. doi: 10.1016/j.mimet.2020.106005 32687865

[18] Walsh AM, Crispie F, O’Sullivan O, Finnegan L, Claesson MJ, Cotter PD. Species classifier choice is a key consideration when analysing low-complexity food microbiome data. Microbiome. 2018;6(1):50. doi: 10.1186/s40168-018-0437-0 29554948 PMC5859664

[19] Franzosa EA, McIver LJ, Rahnavard G, Thompson LR, Schirmer M, Weingart G, et al. Species-level functional profiling of metagenomes and metatranscriptomes. Nat Methods. 2018;15(11):962–8. doi: 10.1038/s41592-018-0176-y 30377376 PMC6235447

[20] Truong DT, Franzosa EA, Tickle TL, Scholz M, Weingart G, Pasolli E, et al. MetaPhlAn2 for enhanced metagenomic taxonomic profiling. Nat Methods. 2015;12(10):902–3. doi: 10.1038/nmeth.3589 26418763

[21] Torondel B, Ensink JH, Gundogdu O, Ijaz UZ, Parkhill J, Abdelahi F, et al. Assessment of the influence of intrinsic environmental and geographical factors on the bacterial ecology of pit latrines. Microb Biotechnol. 2016;9(2):209–23. doi: 10.1111/1751-7915.12334 26875588 PMC4767293

[22] Le Cao KA, Boitard S, Besse P. Sparse PLS discriminant analysis: biologically relevant feature selection and graphical displays for multiclass problems. BMC Bioinformatics. 2011;12:253. doi: 10.1186/1471-2105-12-253 21693065 PMC3133555

[23] Paley S, Parker K, Spaulding A, Tomb JF, O'Maille P, Karp PD. The Omics Dashboard for interactive exploration of gene-expression data. Nucleic Acids Res. 2017;45(21):12113–24. doi: 10.1093/nar/gkx910 29040755 PMC5716103

[24] Haupt E, Knick B, Koschinsky T, Liebermeister H, Schneider J, Hirche H. Oral antidiabetic combination therapy with sulphonylureas and metformin. Diabete Metab. 1991;17(1 Pt 2):224–31. 1936481

[25] Gerritsen J, Fuentes S, Grievink W, van Niftrik L, Tindall BJ, Timmerman HM, et al. Characterization of Romboutsia ilealis gen. nov., sp. nov., isolated from the gastro-intestinal tract of a rat, and proposal for the reclassification of five closely related members of the genus Clostridium into the genera Romboutsia gen. nov., Intestinibacter gen. nov., Terrisporobacter gen. nov. and Asaccharospora gen. nov. Int J Syst Evol Microbiol. 2014;64(Pt 5):1600–16. doi: 10.1099/ijs.0.059543-0 24480908

[26] Brahe LK, Le Chatelier E, Prifti E, Pons N, Kennedy S, Hansen T, et al. Specific gut microbiota features and metabolic markers in postmenopausal women with obesity. Nutr Diabetes. 2015;5:e159. doi: 10.1038/nutd.2015.9 26075636 PMC4491860

[27] Wingfield B, Coleman S, McGinnity TM, Bjourson A. Robust Microbial Markers for Non-Invasive Inflammatory Bowel Disease Identification. IEEE/ACM Trans Comput Biol Bioinform. 2018. doi: 10.1109/TCBB.2018.2831212 29994028

[28] Haro C, Montes-Borrego M, Rangel-Zuniga OA, Alcala-Diaz JF, Gomez-Delgado F, Perez-Martinez P, et al. Two Healthy Diets Modulate Gut Microbial Community Improving Insulin Sensitivity in a Human Obese Population. J Clin Endocrinol Metab. 2016;101(1):233–42. doi: 10.1210/jc.2015-3351 26505825

[29] Wang K, Liao M, Zhou N, Bao L, Ma K, Zheng Z, et al. Parabacteroides distasonis Alleviates Obesity and Metabolic Dysfunctions via Production of Succinate and Secondary Bile Acids. Cell Rep. 2019;26(1):222–35 e5. doi: 10.1016/j.celrep.2018.12.028 30605678

[30] Wei S, Han R, Zhao J, Wang S, Huang M, Wang Y, et al. Intermittent administration of a fasting-mimicking diet intervenes in diabetes progression, restores beta cells and reconstructs gut microbiota in mice. Nutr Metab (Lond). 2018;15:80. doi: 10.1186/s12986-018-0318-3 30479647 PMC6245873

[31] Zhang Y, Cheng Y, Zuo J, Yan L, Li Q, Thring RW, et al. Metformin alleviates insulin resistance in obese-induced mice via remodeling gut microbiota and intestinal metabolites. bioRxiv [Preprint] [Internet]. 2020:[2020.05.26.116715 p.]. Available from: https://www.biorxiv.org/content/biorxiv/early/2020/06/19/2020.05.26.116715.full.pdf.

[32] Palacios T, Vitetta L, Coulson S, Madigan CD, Lam YY, Manuel R, et al. Targeting the Intestinal Microbiota to Prevent Type 2 Diabetes and Enhance the Effect of Metformin on Glycaemia: A Randomised Controlled Pilot Study. Nutrients. 2020;12(7). doi: 10.3390/nu12072041 32660025 PMC7400852

[33] Arboleya S, Watkins C, Stanton C, Ross RP. Gut Bifidobacteria Populations in Human Health and Aging. Front Microbiol. 2016;7:1204. doi: 10.3389/fmicb.2016.01204 27594848 PMC4990546

[34] Makizaki Y, Maeda A, Yamamoto M, Tamura S, Tanaka Y, Nakajima S, et al. Bifidobacterium bifidum G9-1 ameliorates soft feces induced by metformin without affecting its antihyperglycemic action. Biosci Microbiota Food Health. 2020;39(3):145–51. doi: 10.12938/bmfh.2019-022 32775133 PMC7392920

[35] Lord JM, Flight IH, Norman RJ. Metformin in polycystic ovary syndrome: systematic review and meta-analysis. BMJ. 2003;327(7421):951–3. doi: 10.1136/bmj.327.7421.951 14576245 PMC259161

[36] Zheng P, Li Z, Zhou Z. Gut microbiome in type 1 diabetes: A comprehensive review. Diabetes Metab Res Rev. 2018;34(7):e3043. doi: 10.1002/dmrr.3043 29929213 PMC6220847

[37] Weersma RK, Zhernakova A, Fu J. Interaction between drugs and the gut microbiome. Gut. 2020;69(8):1510–9. doi: 10.1136/gutjnl-2019-320204 32409589 PMC7398478

[38] Doumatey AP, Adeyemo A, Zhou J, Lei L, Adebamowo SN, Adebamowo C, et al. Gut Microbiome Profiles Are Associated With Type 2 Diabetes in Urban Africans. Front Cell Infect Microbiol. 2020;10:63. doi: 10.3389/fcimb.2020.00063 32158702 PMC7052266

[39] Yasir M, Angelakis E, Bibi F, Azhar EI, Bachar D, Lagier JC, et al. Comparison of the gut microbiota of people in France and Saudi Arabia. Nutr Diabetes. 2015;5:e153. doi: 10.1038/nutd.2015.3 25915742 PMC4423199

[40] Zhernakova A, Kurilshikov A, Bonder MJ, Tigchelaar EF, Schirmer M, Vatanen T, et al. Population-based metagenomics analysis reveals markers for gut microbiome composition and diversity. Science. 2016;352(6285):565–9. doi: 10.1126/science.aad3369 27126040 PMC5240844

[41] Connors J, Dawe N, Van Limbergen J. The Role of Succinate in the Regulation of Intestinal Inflammation. Nutrients. 2018;11(1). doi: 10.3390/nu11010025 30583500 PMC6356305

[42] Rashid M, Shahzad M, Mahmood S, Khan K. Variability in the therapeutic response of Metformin treatment in patients with type 2 diabetes mellitus. Pak J Med Sci. 2019;35(1):71–6. doi: 10.12669/pjms.35.1.100 30881399 PMC6408638

[43] De Vadder F, Kovatcheva-Datchary P, Zitoun C, Duchampt A, Backhed F, Mithieux G. Microbiota-Produced Succinate Improves Glucose Homeostasis via Intestinal Gluconeogenesis. Cell Metab. 2016;24(1):151–7. doi: 10.1016/j.cmet.2016.06.013 27411015

[44] Pedersen HK, Gudmundsdottir V, Nielsen HB, Hyotylainen T, Nielsen T, Jensen BA, et al. Human gut microbes impact host serum metabolome and insulin sensitivity. Nature. 2016;535(7612):376–81. doi: 10.1038/nature18646 27409811

[45] Leite AZ, Rodrigues NC, Gonzaga MI, Paiolo JCC, de Souza CA, Stefanutto NAV, et al. Detection of Increased Plasma Interleukin-6 Levels and Prevalence of Prevotella copri and Bacteroides vulgatus in the Feces of Type 2 Diabetes Patients. Front Immunol. 2017;8:1107. doi: 10.3389/fimmu.2017.01107 28966614 PMC5605568

[46] De Filippis F, Pasolli E, Tett A, Tarallo S, Naccarati A, De Angelis M, et al. Distinct Genetic and Functional Traits of Human Intestinal Prevotella copri Strains Are Associated with Different Habitual Diets. Cell Host Microbe. 2019;25(3):444–53 e3. doi: 10.1016/j.chom.2019.01.004 30799264

[47] Zhang F, Qiu L, Xu X, Liu Z, Zhan H, Tao X, et al. Bene fi cial effects of probiotic cholesterol-lowering strain of Enterococcus faecium WEFA23 from infants on diet-induced metabolic syndrome in rats. J Dairy Sci. 2017;100(3):1618–28. doi: 10.3168/jds.2016-11870 28041735

[48] Ma Y, Liu J, Hou J, Dong Y, Lu Y, Jin L, et al. Oral administration of recombinant Lactococcus lactis expressing HSP65 and tandemly repeated P277 reduces the incidence of type I diabetes in non-obese diabetic mice. PLoS One. 2014;9(8):e105701. doi: 10.1371/journal.pone.0105701 25157497 PMC4144892

[49] Zeng Z, Yu R, Zuo F, Zhang B, Ma H, Chen S. Recombinant Lactococcus lactis expressing bioactive exendin-4 to promote insulin secretion and beta-cell proliferation in vitro. Appl Microbiol Biotechnol. 2017;101(19):7177–86. doi: 10.1007/s00253-017-8410-6 28828521

[50] Yamashita M, Okubo H, Kobuke K, Ohno H, Oki K, Yoneda M, et al. Alteration of gut microbiota by a Westernized lifestyle and its correlation with insulin resistance in non-diabetic Japanese men. J Diabetes Investig. 2019;10(6):1463–70. doi: 10.1111/jdi.13048 30901505 PMC6825921

[51] Walter J, Martinez I, Rose DJ. Holobiont nutrition: considering the role of the gastrointestinal microbiota in the health benefits of whole grains. Gut Microbes. 2013;4(4):340–6. doi: 10.4161/gmic.24707 23645316 PMC3744518

[52] Ganesan K, Chung SK, Vanamala J, Xu B. Causal Relationship between Diet-Induced Gut Microbiota Changes and Diabetes: A Novel Strategy to Transplant Faecalibacterium prausnitzii in Preventing Diabetes. Int J Mol Sci. 2018;19(12). doi: 10.3390/ijms19123720 30467295 PMC6320976

[53] Siljander H, Honkanen J, Knip M. Microbiome and type 1 diabetes. EBioMedicine. 2019;46:512–21. doi: 10.1016/j.ebiom.2019.06.031 31257149 PMC6710855

[54] Wexler AG, Goodman AL. An insider's perspective: Bacteroides as a window into the microbiome. Nat Microbiol. 2017;2:17026. doi: 10.1038/nmicrobiol.2017.26 28440278 PMC5679392

[55] Wang Y, Luo X, Mao X, Tao Y, Ran X, Zhao H, et al. Gut microbiome analysis of type 2 diabetic patients from the Chinese minority ethnic groups the Uygurs and Kazaks. PLoS One. 2017;12(3):e0172774. doi: 10.1371/journal.pone.0172774 28328990 PMC5362050

[56] Gurung M, Li Z, You H, Rodrigues R, Jump DB, Morgun A, et al. Role of gut microbiota in type 2 diabetes pathophysiology. EBioMedicine. 2020;51:102590. doi: 10.1016/j.ebiom.2019.11.051 31901868 PMC6948163

[57] Zhang X, Shen D, Fang Z, Jie Z, Qiu X, Zhang C, et al. Human gut microbiota changes reveal the progression of glucose intolerance. PLoS One. 2013;8(8):e71108. doi: 10.1371/journal.pone.0071108 24013136 PMC3754967

[58] Jones AG, Lonergan M, Henley WE, Pearson ER, Hattersley AT, Shields BM. Should Studies of Diabetes Treatment Stratification Correct for Baseline HbA1c? PLoS One. 2016;11(4):e0152428. doi: 10.1371/journal.pone.0152428 27050911 PMC4822872

[59] Ma W, Chen J, Meng Y, Yang J, Cui Q, Zhou Y. Metformin Alters Gut Microbiota of Healthy Mice: Implication for Its Potential Role in Gut Microbiota Homeostasis. Front Microbiol. 2018;9:1336. doi: 10.3389/fmicb.2018.01336 29988362 PMC6023991

[60] Rosario D, Benfeitas R, Bidkhori G, Zhang C, Uhlen M, Shoaie S, et al. Understanding the Representative Gut Microbiota Dysbiosis in Metformin-Treated Type 2 Diabetes Patients Using Genome-Scale Metabolic Modeling. Front Physiol. 2018;9:775. doi: 10.3389/fphys.2018.00775 29988585 PMC6026676

[61] Porter KM, Ward M, Hughes CF, O'Kane M, Hoey L, McCann A, et al. Hyperglycemia and Metformin Use Are Associated With B Vitamin Deficiency and Cognitive Dysfunction in Older Adults. J Clin Endocrinol Metab. 2019;104(10):4837–47. doi: 10.1210/jc.2018-01791 30920623

[62] Wakeman M, Archer DT. Metformin and Micronutrient Status in Type 2 Diabetes: Does Polypharmacy Involving Acid-Suppressing Medications Affect Vitamin B12 Levels? Diabetes Metab Syndr Obes. 2020;13:2093–108. doi: 10.2147/DMSO.S237454 32606868 PMC7308123

[63] Cabreiro F, Au C, Leung KY, Vergara-Irigaray N, Cocheme HM, Noori T, et al. Metformin retards aging in C. elegans by altering microbial folate and methionine metabolism. Cell. 2013;153(1):228–39. doi: 10.1016/j.cell.2013.02.035 23540700 PMC3898468

[64] Olgun A. "Metformin-resistant" folic acid producing probiotics or folic acid against metformin's adverse effects like diarrhea. Med Hypotheses. 2017;106:33–4. doi: 10.1016/j.mehy.2017.07.009 28818268

[65] Asquith M, Rosenbaum JT. The interaction between host genetics and the microbiome in the pathogenesis of spondyloarthropathies. Curr Opin Rheumatol. 2016;28(4):405–12. doi: 10.1097/BOR.0000000000000299 27152700

