# Supplementary material for: Baseline gut microbiome composition predicts metformin therapy short-term efficacy in newly diagnosed type 2 diabetes patients
Source: PLoS One. 2020 Oct 30;15(10):e0241338. doi: 10.1371/journal.pone.0241338 (PMC7598494; doi:10.1371/journal.pone.0241338)
Supplement: S1 Text — (DOCX) [file pone.0241338.s001.docx]

S1 Text. List of inclusion/exclusion criteria

1. List of inclusion/exclusion criteria for the cohort oh healthy individuals

- 1. Inclusion criteria:
  2. A healthy individual:

(1) With no known illnesses at the time of application to this study that could affect the result of the study;

(2) Whose body characteristics are within the healthy reference interval (e.g., BMI range is 18.5 – 29.9);

(3) Whose mental state allows him to understand the research process, and give a legal consent for the participation in it;

(4) Whose physical state allows complying with the needs of the study protocol.

- 1. Age: 18 – 64 years;
  2. European descent;
  3. Both males and females with reproductive potential match the contraception requirements of the study protocol.
  4. Prior to the study-related procedures, the consent of a person's participation in the clinical trial is received by submitting a signed and dated informed consent document.
  5. Exclusion criteria:
  6. Hypersensitivity to any of the components in *Metforal* 850mg;
  7. Use of any medication that is not compatible with *Metforal* 850mg therapy (according to *Metforal* description);
  8. Pregnancy or lactation;
  9. Diagnosis of type 1 or type 2 *diabetes mellitus*, pancreatogenic diabetes, impaired glucose tolerance (evaluated by HbA_1c_ and fasting glucose levels);
  10. Polycystic ovary syndrome;
  11. Chronical gastrointestinal, oncological, or autoimmune diseases;
  12. Renal failure or dysfunction (evaluated by glomerular filtration rate - Cockcroft-Gault formula);
  13. Liver dysfunction (ALAT results are not in the reference interval) or alcoholism;
  14. Acute conditions with possible effects on kidney functions (dehydration, severe infection, shock);
  15. Acute or chronical diseases that could cause tissue hypoxia, (e.g., heart or breathing failure, recent myocardial infarct, shock);
  16. Diarrhea during the week before the study;
  17. Previous long term use of metformin;
  18. Use of any of the following medications in the past two months:
      1. Antibiotics;
      2. Pharmaceutical-grade probiotics;
      3. Proton pump inhibitors (e.g. omeprazole, lansoprazole, pantoprazole, etc.);
      4. Immunosuppressive drugs (methotrexate, etc.);
      5. Corticosteroids (e.g. cortisone, hydrocortisone, prednisolone, etc.);
  19. Concurrently to the study, any radiologic procedures involving intravascular administration of iodinated contrast materials are intended.

2. List of inclusion/exclusion criteria for the OPTIMED cohort

2.1. Inclusion criteria

- - - 1. Newly diagnosed type 2 diabetes mellitus and initiation of oral antidiabetic therapy;
      2. Previous diagnosis of type 2 diabetes mellitus and no oral antidiabetic or insulin therapy used in the previous three months;
      3. Newly diagnosed patients for glycemic control for an acute on-site intensive insulin therapy up to five days, continued afterwards;
      4. Patients unavailable and not optimized in drug trials;
      5. Age of 18;
      6. Patients meeting the diagnostic criteria for type 2 diabetes mellitus:
    1. Fasting blood glucose ≥7 mmol / l;
    2. Blood glucose two hours after OGTT with 75 g glucose ≥11.1 mmol / l.
       1. Prior to the study-related procedures, the consent of a person's participation in the clinical trial is received by submitting a signed and dated informed consent document.

2.2. Exclusion criteria:

1. Use of peroral antidiabetic therapy;
2. Use of Type 2 *diabetes mellitus* insulin therapy;
3. Pregnancy.

ALAT - alanine aminotransferase; HbA_1c_ - hemoglobin A1c; OGTT – oral glucose tolerance test.
