# Supplementary material for: Baseline gut microbiome composition predicts metformin therapy short-term efficacy in newly diagnosed type 2 diabetes patients
Source: PLoS One. 2020 Oct 30;15(10):e0241338. doi: 10.1371/journal.pone.0241338 (PMC7598494; doi:10.1371/journal.pone.0241338)
Supplement: S2 Text — (DOCX) [file pone.0241338.s002.docx]

S2 Text. Methodological description of Validation cohort sample and data processing.

Inclusion/exclusion criteria, sample collection guidelines and design for Validation cohort were the same as for the OPTIMED cohort. Briefly:

1. **Sample collection:** Stool samples were collected in two aliquots at pre-determined time points during the study. Samples were coded as follows: M0 – before metformin treatment, and M7d – 7 days after starting the therapy. All samples were collected by participants at home, using sterile collection tubes without buffer (collection date and time were marked). Within 24 hours participants delivered samples to the closest clinical or research laboratory where samples were frozen at −80°C (delivery time was registered).

Blood samples for biochemical/hematological analysis (conducted in a certified clinical laboratory) to evaluate inclusion/exclusion criteria and obtain relevant clinical data were collected from participants within both cohorts. Samples were collected in the fasting state before starting metformin administration. In addition, a repeated biochemical/hematological analysis was performed three months later (follow-up coded as a time point M3m).

1. **DNA extraction, shotgun-metagenome library preparation and sequencing:** Microbial DNA was extracted using the FastDNA Spin Kit for Soil (MP Biomedicals) in line with to the manufacturer's instructions. Further shotgun metagenomic library preparation was done by fragmenting the DNA at 400 bp (Covaris) and following the manual of the MGIEasy Universal DNA Library Prep Set (MGI Tech Co. Ltd). That included the following sample processing steps: (1) end repair and A-tailing after the physical fragmentation, (2) Barcode Adapter ligation and clean-up with MGIEasy DNA Clean Beads, (3) amplification and clean-up. After that, the appropriate count of libraries (to obtain the planned read count) were pooled and each pool was normalized to 330 ng in a volume of 48 μl. The pooled libraries were further annealed and circularized with splint oligo. Further, DNA nanoballs were created with rolling circle amplification. The end-products were sequenced using DNBSEQ-G400RS sequencing platform (~30000000 reads/sample).
2. **Sequence analysis and statistics:** Raw data from the sequencer were processed as follows: adapters were removed with cutadapt 1.16, sequences were trimmed with Trimmomatic v0.38 (5bp window, quality threshold = 20, average quality = 20, minimal length = 75), mapping was performed with bowtie2-2.3.5.1 using Homo sapiens genome Ensembl GRCh38 release-90 reference to remove host DNA sequences. Composition and functionality from the remaining sequences of gut microbiome samples were analyzed using the HUMAnN2 pipeline, and taxonomic data were obtained with MetaPhlAn2, analyses were performed with default parameters.
