# Supplementary figures and images for: Baseline gut microbiome composition predicts metformin therapy short-term efficacy in newly diagnosed type 2 diabetes patients
Source: PLoS One. 2020 Oct 30;15(10):e0241338. doi: 10.1371/journal.pone.0241338 (PMC7598494; doi:10.1371/journal.pone.0241338)

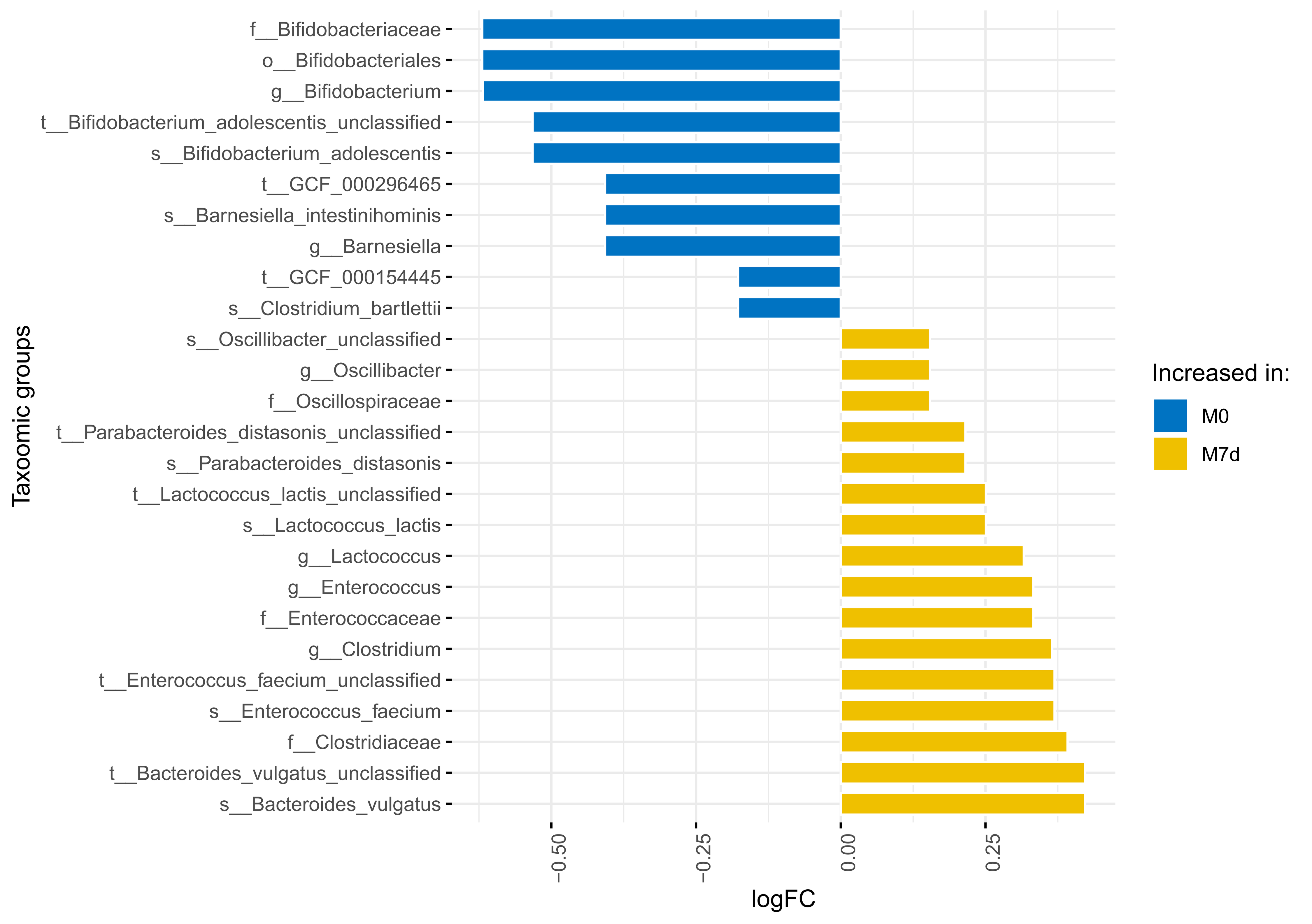

Supplement: S1 Fig — Differentiating feature analysis was carried out with limma+voom, adjusted p-value cut-off = 0.05. Samples coded as follows: M0 –before starting metformin treatment (blue, negative logFC), and M7d – 7 days after the first intake of metformin (yellow, positive logFC). logFC–log fold change. (TIF) [file pone.0241338.s003.tif]

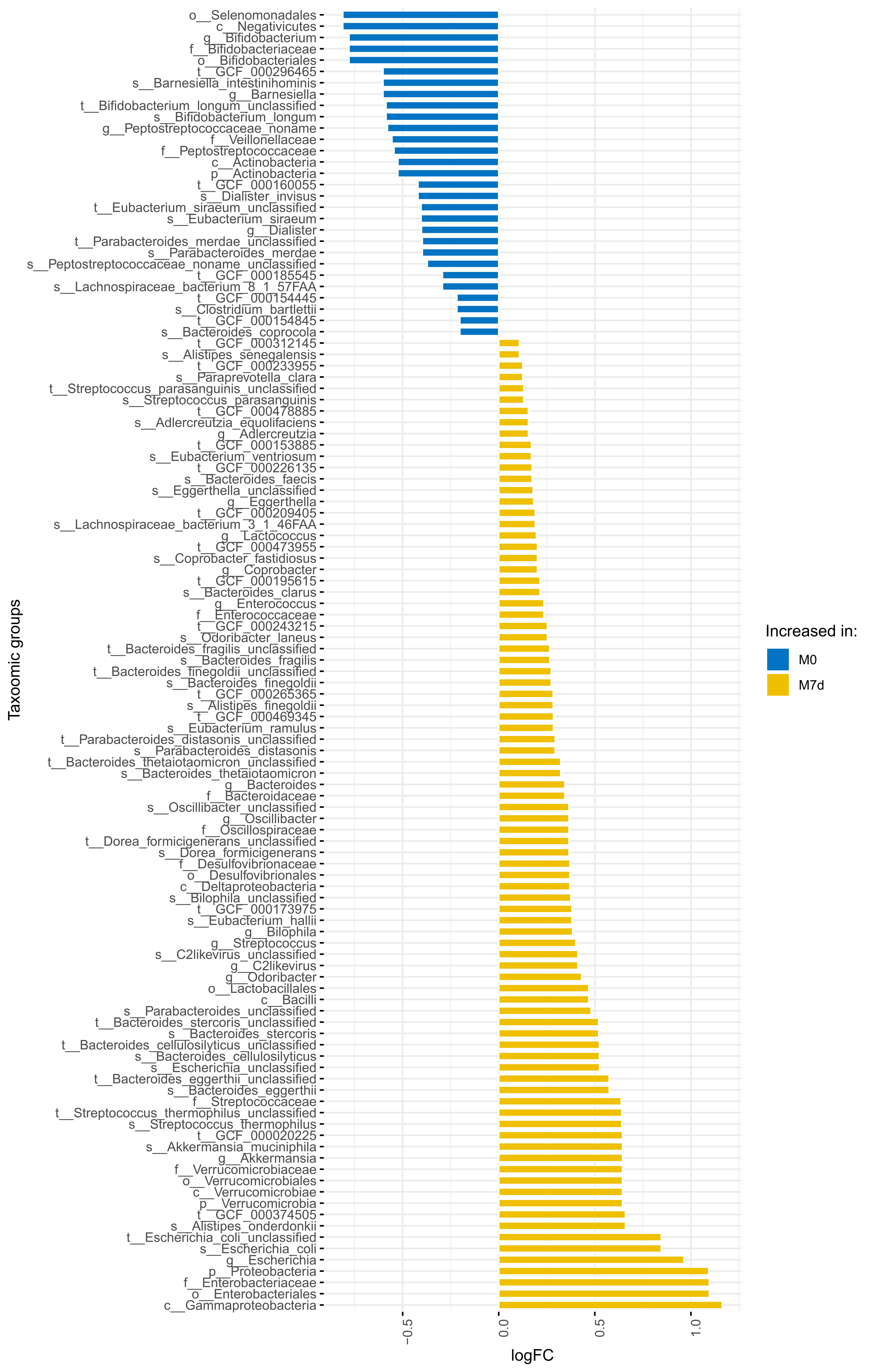

Supplement: S2 Fig — Differentiating feature analysis was carried out with limma+voom, adjusted p-value cut-off = 0.05. Samples coded as follows: M0 –before starting metformin treatment (blue, negative logFC), and M7d – 7 days after the first intake of metformin (yellow, positive logFC). logFC–log fold change. (TIF) [file pone.0241338.s004.tif]

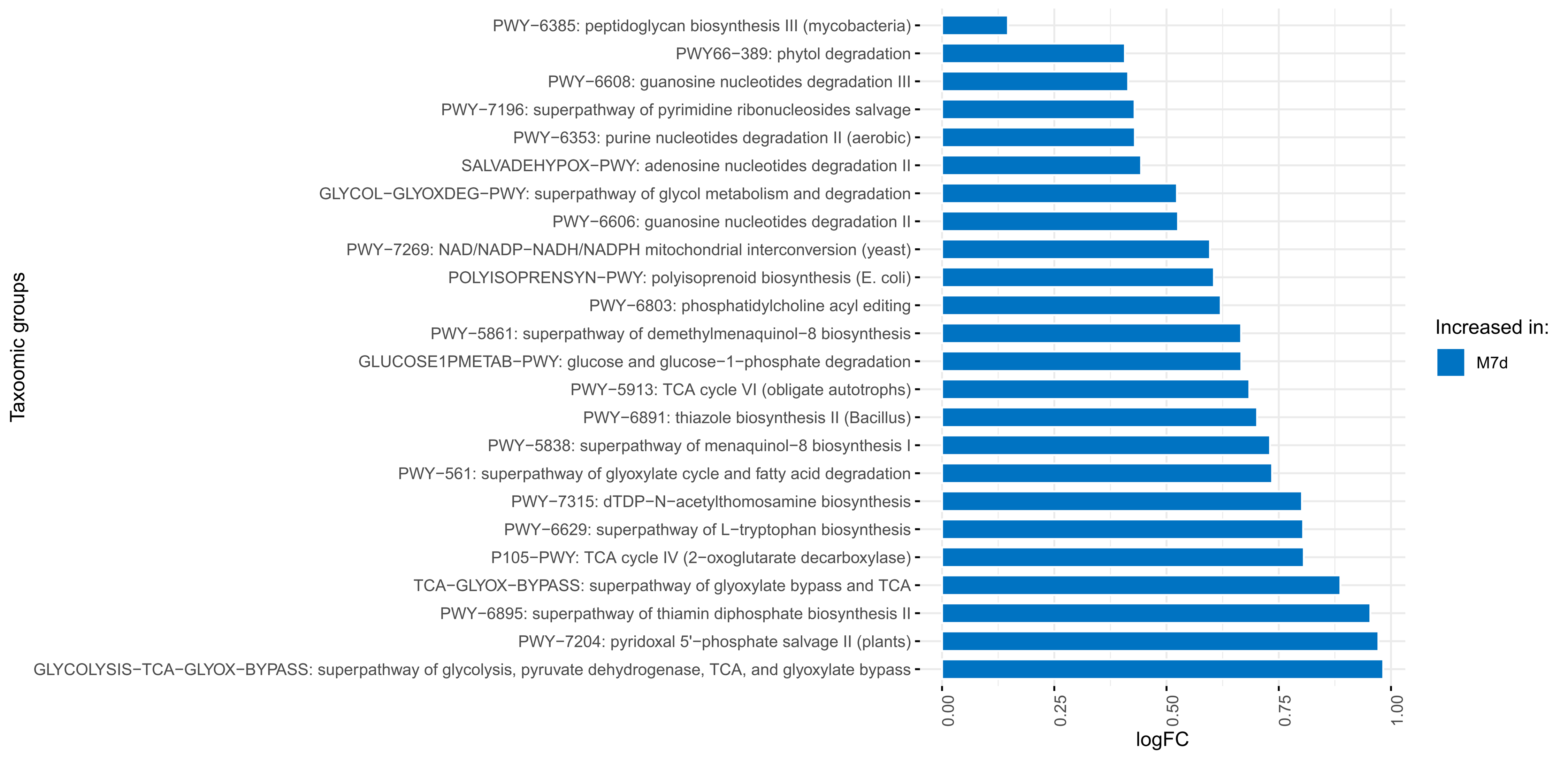

Supplement: S3 Fig — Differentiating feature analysis was carried out with limma+voom, adjusted p-value cut off = 0.05. Samples coded as follows: M7d – 7 days after the first intake of metformin (blue, positive logFC). logFC–log fold change. (TIF) [file pone.0241338.s005.tif]

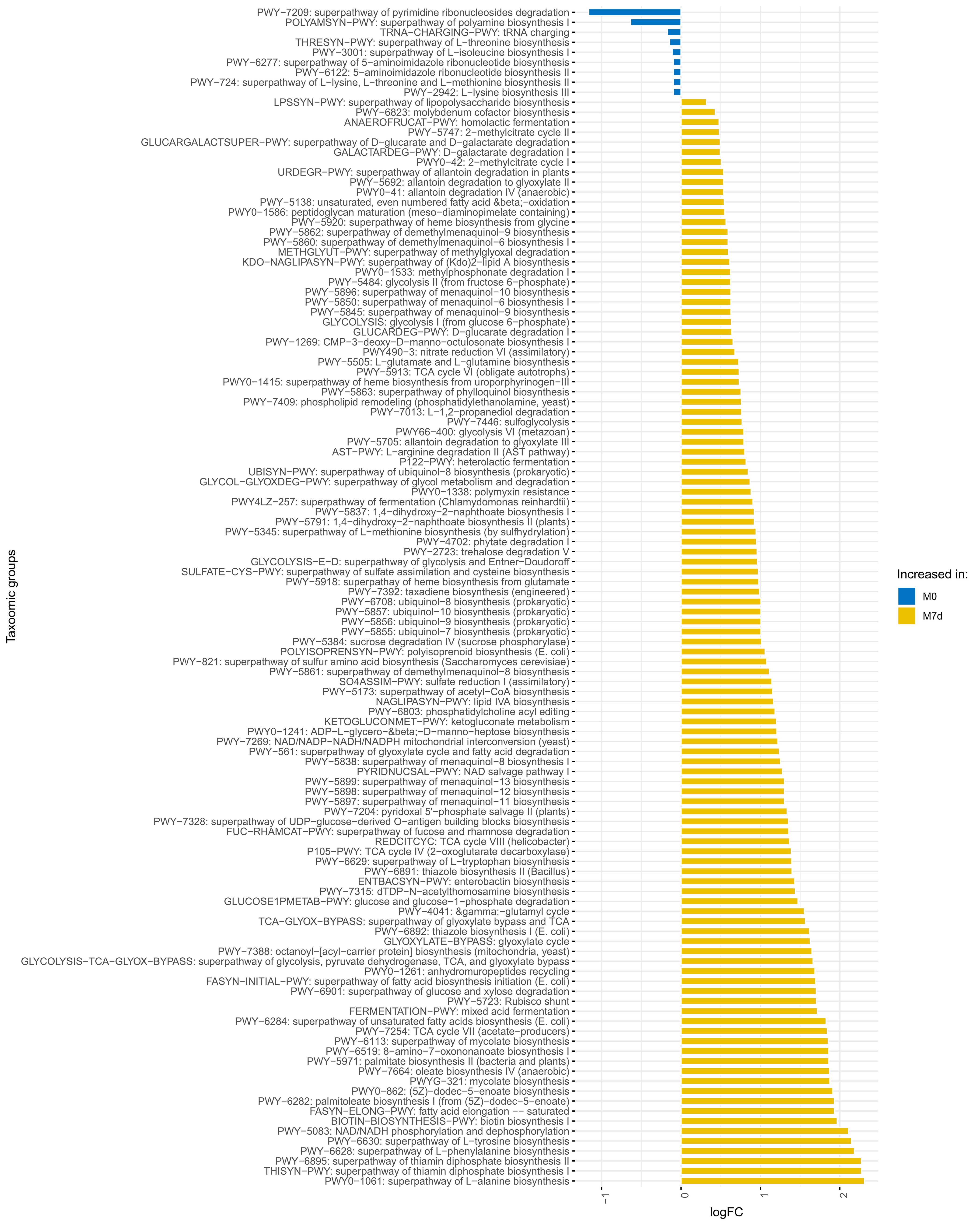

Supplement: S4 Fig — Differentiating feature analysis was carried out with limma+voom, adjusted p-value cut off = 0.05. Samples coded as follows: M0 –before starting metformin treatment (blue, negative logFC), and M7d – 7 days after the first intake of metformin (yellow, positive logFC). logFC–log fold change. (TIF) [file pone.0241338.s006.tif]
