# Supplementary material for: Baseline gut microbiome composition predicts metformin therapy short-term efficacy in newly diagnosed type 2 diabetes patients
Source: PLoS One. 2020 Oct 30;15(10):e0241338. doi: 10.1371/journal.pone.0241338 (PMC7598494; doi:10.1371/journal.pone.0241338)
Supplement: S1 Table — (DOCX) [file pone.0241338.s007.docx]

S1 Table – Taxonomic groups with VIP score >1.5 in at least one of the first two components from sPLS‑DA analysis.

| Taxonomic group | Component 1 | Component 2 |
| --- | --- | --- |
| g_*Dialister* | 4.04 | 3.61 |
| s_*Enterococcus faecium* | 3.80 | 3.40 |
| t_*Enterococcus faecium* unclassified | 3.80 | 3.40 |
| g_*Lactococcus* | 3.37 | 3.01 |
| s_*Lactococcus lactis* | 3.22 | 2.87 |
| t_*Lactococcus lactis* unclassified | 3.22 | 2.87 |
| g_*Odoribacter* | 3.16 | 2.85 |
| s_*Roseburia* unclassified | 3.10 | 2.77 |
| s_*Odoribacter* unclassified | 3.01 | 2.70 |
| s_*Peptostreptococcaceae noname* unclassified | 2.90 | 2.59 |
| s_*Bacteroides coprocola* | 2.72 | 2.43 |
| t_GCF 000154845 | 2.72 | 2.43 |
| s_*Bacteroides vulgatus* | 2.69 | 2.45 |
| t_*Bacteroides vulgatus* unclassified | 2.69 | 2.45 |
| s_*Bifidobacterium* *bifidum* | 2.60 | 2.42 |
| t_*Bifidobacterium bifidum* unclassified | 2.60 | 2.42 |
| p_*Bacteroidetes* | 2.51 | 2.28 |
| c_*Bacteroidia* | 2.51 | 2.28 |
| o_*Bacteroidales* | 2.51 | 2.28 |
| f_*Bacteroidaceae* | 2.45 | 2.21 |
| g_*Bacteroides* | 2.45 | 2.21 |
| g_*Ruminococcus* | 2.35 | 2.09 |
| s_*Bacteroides cellulosilyticus* | 2.06 | 1.84 |
| t_*Bacteroides cellulosilyticus* unclassified | 2.06 | 1.84 |
| g_*Coprobacter* | 2.05 | 1.83 |
| s_*Coprobacter fastidiosus* | 2.05 | 1.83 |
| t_GCF 000473955 | 2.05 | 1.83 |
| s_*Lachnospiraceae bacterium* 5_1_63FAA | 1.98 | 1.77 |
| t_GCF 000185525 | 1.98 | 1.77 |
| s_*Ruminococcus bromii* | 1.89 | 1.69 |
| t_GCF 000209875 | 1.89 | 1.69 |
| g_*Subdoligranulum* | 1.73 | 1.55 |
| s_*Subdoligranulum* unclassified | 1.73 | 1.55 |
| s_*Bacteroides finegoldii* | 1.71 | 1.93 |
| t_*Bacteroides finegoldii* unclassified | 1.71 | 1.93 |
| s_*Bacteroides fragilis* | 1.70 | 1.52 |
| t_*Bacteroides fragilis* unclassified | 1.70 | 1.52 |
| s_*Eubacterium siraeum* | 1.57 | 1.40 |
| t_*Eubacterium siraeum* unclassified | 1.57 | 1.40 |
| p_*Proteobacteria* | 1.05 | 1.79 |
| g_*Erysipelotrichaceae noname* | 0.21 | 1.54 |
| s_*Prevotella stercorea* | 0.00 | 1.93 |
| t_GCF 000235885 | 0.00 | 1.93 |
